# Deathly Silent: Exploring the Global Lack of Data Relating to Stranded Cetacean Euthanasia

**DOI:** 10.3390/ani11051460

**Published:** 2021-05-19

**Authors:** Rebecca M. Boys, Ngaio J. Beausoleil, Emma L. Betty, Karen A. Stockin

**Affiliations:** 1 Cetacean Ecology Research Group, School of Natural and Computational Sciences, College of Sciences, Massey University, Private Bag 102-904, Auckland 0632, New Zealand; e.l.betty@massey.ac.nz; 2 Animal Welfare Science and Bioethics Centre, School of Veterinary Science, College of Sciences, Massey University, Private Bag 11-222, Palmerston North 4442, New Zealand; N.J.Beausoleil@massey.ac.nz

**Keywords:** euthanasia, welfare, strandings, death, insensibility, marine mammals, cetacean

## Abstract

**Simple Summary:**

Cetacean strandings are frequent in occurrence and are likely to become even more common globally because of the effects of escalating anthropogenic activities. Due to the compromised state of stranded animals, euthanasia is often recommended or required. However, current knowledge and implementation of euthanasia methods remain highly variable, with limited data on the practicalities and welfare impacts of procedures. This study sought to evaluate the available published data on cetacean euthanasia in order to highlight significant knowledge gaps and provide direction to improve the welfare of stranded cetaceans. Data from the peer-reviewed literature and published reports were analysed, and significant knowledge gaps highlighted. Two main euthanasia methods, chemical and ballistics, were reported, with few details provided on the specific application of these. Few data were available about time to death/insensibility, parameters commonly required to assess the welfare impacts of killing methods. Overall, the findings highlight the lack of available information on cetacean euthanasia and suggest avenues for future work to improve welfare through the use of appropriate methods and increased data collection.

**Abstract:**

The compromised state of stranded cetaceans means that euthanasia is often required. However, current knowledge and implementation of euthanasia methods remain highly variable, with limited data on the practicalities and welfare impacts of procedures. This study evaluated the available published data on cetacean euthanasia, highlighting knowledge gaps and providing direction to improve stranded cetacean welfare. A total of 2147 peer-reviewed articles describing marine mammal euthanasia were examined. Of these 3.1% provided details on the method used, with 91% employing chemical methods. Two countries, the United Kingdom (UK) and New Zealand (NZ), provided euthanasia reports to the International Whaling Commission (IWC) between 2007 and 2020. Methods employed were reported for 78.3% and 100% of individual cetaceans euthanised in the UK and NZ, respectively. In the UK, chemical euthanasia was most common (52%), whilst in NZ only ballistics methods were used. Few data were available about time to death/insensibility (TTD); 0.5% of peer-reviewed articles provided TTD, whilst TTD was reported for 35% of individuals in the UK and for 98% in NZ. However, IWC reports lacked detail on how death/insensibility were assessed, with multiple individuals “presumed instantly” killed. Overall, the findings highlight the lack of available information on cetacean euthanasia, and suggest increased data collection and the application of appropriate methods to improve welfare.

## 1. Introduction

Cetacean strandings are predicted to increase in the future, as global marine mammal health continues to decline [1]. Factors contributing to the decline include climate change [2,3] and increasing anthropogenic activities [4,5]. The characteristics of individual animals found stranded can vary significantly, with some animals appearing outwardly healthy, while others range from being clinically ill to moribund or dead [6]. Despite their compromised state and a lack of empirical evidence to support rescue attempts, most live cetacean stranding events will involve human interventions driven by a societal desire to “rescue” animals by attempting to refloat them [7]. Indeed, some intervention decisions have led to significantly debilitated individuals being refloated, enduring prolonged suffering and leading to further re-strandings [8,9,10,11,12].

Most live stranding events involve compromised individuals with notable injuries and/or illness. Therefore, the stranding itself along with subsequent rescue attempts will likely compromise both animal welfare and survival, in addition to hampering the achievements of conservation goals. Consequently, in many cases the refloatation or rehabilitation of such debilitated animals is not feasible or desirable, and killing may be required to end suffering [13,14]. However, several factors elicit controversy when it comes to this decision-making. These include a lack of detailed guidelines and protocols for cetacean euthanasia; the absence of quantitative studies underpinning current protocols; and a range of socio-economic, traditional and, in some cases, religious beliefs [15]. To ensure animal welfare compromise is minimised, reliable methods for the humane killing of cetaceans will be increasingly required. To be viable, such methods need to be safe for the personnel involved, humane, publicly accepted and cost effective [16,17].

The word euthanasia comes from the Greek, meaning good (eu) and death (thanatos). According to the American Veterinary Medical Association, euthanasia is used to describe the ending of the life of an individual animal that minimises distress and pain [18]. In the case of stranded marine mammals, it should also include that it is the humane ending of life for an animal that is otherwise suffering. Therefore, techniques employed should result in a rapid loss of consciousness followed by cardiac arrest and the loss of brain function. Notably, methods should further minimise the level of anxiety or distress experienced by the animal prior to loss of consciousness [19].

Euthanasia methods applied to stranded cetaceans remain highly variable, with a lack of sufficient empirical data to support standardised procedures [17]. Multiple approaches have been applied, which can be broadly characterised into chemical (parenteral injection and inhalation) and physical (ballistics, explosives and exsanguination). However, specific details such as the chemical and quantity employed, route of administration, firearm calibre and projectile characteristics, amount of explosive charge and artery cut can vary significantly. The most appropriate method will also vary depending upon the taxa stranded and features of the stranding event, such as the location and the presence of trained personnel.

Though chemical euthanasia is common in captive settings, and may be rapid and effective if executed correctly, the logistical complexity in stranding situations often makes it a non-viable option [20]. This is particularly the case in mass stranding scenarios or when dealing with large species. This is because the substances used are often controlled, requiring veterinary personnel for administration, and are required in relatively large quantities [17]. On the other hand, when carried out appropriately, physical methods such as ballistics can cause instantaneous death [21], as they target the brain directly. However, these may be complicated by the unique cranial anatomy of cetaceans, which may lead to severe wounding rather than death if employed inappropriately [22,23].

Following euthanasia, verification of death is vital in order to assess the humaneness of the method by examining the duration and intensity of suffering before the animal becomes permanently insensible [18,19]. The most commonly employed parameter to quantify humaneness is time to death (TTD) or insensibility [24,25]. However, assessing death or insensibility in cetaceans can be complicated. The thick blubber layer means that reliable criteria such as the absence of a heartbeat [26] cannot always be consistently employed. Although there continue to be discrepancies in the methods for assessing insensibility and death in cetacea, a number of criteria are universally recommended. These include lack of jaw tone, absence of eye reflexes (menace, palpebral and corneal), fixed dilated pupils, lack of response to stimuli around blowhole, no capillary refill time and ocular/skin temperature differential [27,28].

Currently, there are few studies that provide information on marine mammal euthanasia [14,16,23,29,30,31,32,33,34]. Generally, there is little information on how often stranding events end in euthanasia, and in such cases, how euthanasia is actually achieved. Furthermore, there is also a lack of information on TTD in such cases and the criteria used to assess death or insensibility, necessary to understand welfare impacts, are often not reported. The aim of this study was to investigate the currently available information regarding cetacean euthanasia methods and efficacy based on TTD to highlight knowledge gaps and suggest directions for improving the knowledge and welfare of stranded cetaceans. This was achieved by 1) examining the peer-reviewed literature at a global scale for articles pertaining to marine mammal euthanasia, 2) investigating unpublished data at a global scale via countries reporting cetacean deaths to the International Whaling Commission (IWC) and 3) investigating historical data collected by New Zealand (NZ), a country known for its high cetacean stranding incidence.

## 2. Materials and Methods

### 2.1. Peer-Reviewed Literature

We examined current international practice for marine mammal euthanasia to assess what information is available, and to examine discrepancies between methods in the amount of information available as well as reported time to death (TTD). A search of the English language peer-reviewed literature was carried out using Web of Science and Google Scholar for the time period January 1930 to September 2020. We searched for publications involving marine mammals that had the word euthanasia or killing (or their derivatives) in the title, keywords, abstract and anywhere in the main text of the article (TS = (Euthan*) OR (Kill*) AND TS = (porpoise* OR dolphin* OR whale* OR manatee* OR dugong* OR otter* OR “polar bear*” OR cetacean* OR pinniped* OR seal* OR “sea lion*” OR “marine mammal” *NOT* TOPIC: (sealant*) *NOT* TOPIC: (sealer*) *NOT* TOPIC: (construct*)). Publications that contained relevant words were compiled into a database using Microsoft Excel, in which duplicates were detected and removed manually. Furthermore, articles that related to hunting-only of marine mammals were removed. Each article was then categorised based on the taxon/species involved, with references to freshwater species further removed. Next, we extracted publications that contained some information on the methods applied. In the final stage we extracted publications that provided an estimated TTD. These two categories were based on either cessation of the heart or loss of all conscious reflexes [27,28]. The collated data (Tables S1 and S2) were then used to investigate how many different methods were applied based on species/taxon. Taxa were separated into delphinid, delphinid (blackfish), mysticete, odontocete (other than delphinid), pinniped, mustelid and ursid (polar bears). No peer-reviewed studies were found that included sirenians in relation to euthanasia. The data were also used to investigate how often TTD or insensibility data were reported, what criteria were reported to assess this and whether there were differences in TTD or insensibility based on the method applied.

### 2.2. International Whaling Commission (IWC) Data

The IWC encourages its member states (n = 88 as of 2020) to submit information on any individual cetacean-killing event including TTD [35]. While these reported data are submitted to the Whale Killing and Welfare Subcommittee and are available online within a public archive (https://archive.iwc.int/pages/home.php?login=true (accessed on 15 September 2020), they are not published in the scientific literature or summarized in any commission report.

Over the period 2007–2020, six member states (Alaska, Greenland, New Zealand, Russia, St Vincent and Grenadines and the United Kingdom) reported data on cetacean deaths, with most related to hunting (n = 4, Alaska, Greenland, Russia and St Vincent and Grenadines). The remaining two nations, New Zealand (NZ) and the United Kingdom (UK), have reported on the killing of individual cetaceans at stranding events for the purpose of ending suffering (euthanasia). The reports available span 13 years (2007–2020) for NZ and 4 years (2014–2018) for the UK.

The IWC archives were data-mined specifically to extract information about cetacean euthanasia events, including (1) methods of euthanasia applied, (2) TTD or insensibility, (3) taxa euthanised and (4) stranding type (single or mass). Given the anatomical variability of species reported, cetacea were split into five broad categories (mysticete, ziphiid, delphinid, phocoenid, delphinid (blackfish) and kogiid; see Tables S3–S5). The kogiids were placed into their own category due to their anatomical differences from the other taxa, including their asymmetrical skull, concave cranium, small spermaceti organ and blowhole placement [36,37], which may affect anatomical landmarks used for euthanasia via ballistics. Similarly, ziphiids were considered a separate category due to their unusual skull structure, including the thickened irregular nasal sinuses, variation in vertex and ultra-dense tissues [38,39], which may affect euthanasia via ballistics.

### 2.3. New Zealand—Historical Records

In addition to the data that NZ has reported to the IWC (2007–2020), opportunistic data on individual cetacean euthanasia prior to the initiation of these reports (1991–2006) has also been collected by the Department of Conservation (DOC). This data set was examined to extract additional information on (i) methods of euthanasia applied, (ii) TTD or insensibility and (iii) taxa euthanised (as detailed previously).

All data collected from the IWC and historical records were broken down into categories of year, species and the total number of individuals euthanised. The different methods applied for euthanasia were then related to each category, where available detailed information on firearm calibre and injection route was noted. The total number of individuals euthanised via each detailed method and the related TTD or insensibility data was then added. Any further data provided, such as projectile characteristics and numbers of shots for ballistics, and chemical solution and dosage for chemical euthanasia, were also collated into this database. Finally, the species were collated into taxa categories to enable the examination of any differences in taxa being euthanised, methods being applied and TTD or insensibility reported.

## 3. Results

### 3.1. Peer-Reviewed Literature

An examination of English-language peer-reviewed literature spanning 70 years (January 1930 to September 2020) revealed that articles pertaining to marine mammals and euthanasia have only been published since 1980. In the last 40 years, a total of 2147 articles referring to marine mammals (cetacea, pinniped, mustelid and ursid polar bear) in the context of euthanasia have been published. Only 3.1% (n = 66) of those articles stated the euthanasia method applied (chemical or physical), with 10.4% (n = 7) of these discussing the euthanasia of multiple individuals where a number of methods were employed, including chemical injection, chemical inhalation, ballistics and exsanguination.

Of those articles that reported methods, chemical euthanasia was most common (91%, n = 60), followed by one of the physical methods, ballistics (12%, n = 8), with one article describing the use of both methods. For chemical euthanasia, the route of parenteral injection was reported for 73% (n = 44) of cases in which the method of euthanasia was reported. In some of these articles (n = 11), multiple routes were described due to their reporting of euthanasia for several individual animals. These routes included intra-muscular (IM; n = 15), intra-venous (IV; n = 35), intra-cardiac (IC; n = 7), intra-hepatic (IH; n = 1), intra-peritoneal (IP; n = 2), intra-thoracic (IT; n = 1) and retrobulbar (n = 1), and three articles also described inhalation. The most common chemical euthanasia agents were barbiturates (n = 35). A number of articles described the use of sedatives prior to euthanasia, including acepromazine, medetomidine, midazolam, xylazine and diazepam, with two articles describing their use alone as sufficient to achieve euthanasia.

Firearm calibre was reported in 75% (n = 6) of ballistics cases, with six differing calibres reported. Projectile characteristics featured in only 38% (n = 3) of these cases, with all three being different projectiles. Four of the articles also provided detail on the orientation at which the firearm was shot, being either dorso-ventral (n = 4) or lateral (n = 2). One case provided detail on the method of explosives used and detailed the quantities, type and location of set charges.

Time to death was detailed in very few articles (0.5%, n = 10). Nine cases (n = 9) that reported TTD had employed chemical methods for euthanasia, and only one (10%), which reported instantaneous death, had employed the physical method of explosives. TTD following chemical injection varied from 5 min to 49.7 h (median = 48 min, mean = 4.7 h, SD = 13 h). Eight of the ten studies reported criteria used to confirm death including loss of palpebral, corneal and tongue reflexes, absence of respiration, absence of all vital signs, cessation of cardiac activity (movements and sound) and relaxation of jaw muscles.

Pinnipeds were the focus taxa of euthanasia literature that detailed methods (53.7%, n = 36), followed by delphinids (blackfish) (16.4%, n = 11) and mysticetes (16.4%, n = 11). In contrast, the reporting of TTD primarily focused on mysticetes (60%, n = 6), delphinids (20%, n = 2) and other odontocetes (20%, n = 2).

### 3.2. IWC Data

Of the 88 member nations, only two (UK and NZ) submitted individual stranded cetacean euthanasia data to the IWC as part of their National Progress reporting to the annual Scientific Commission meeting (Tables S3–S5). In addition, the DOC in NZ also collected data on individual stranded cetacean euthanasia (1991–2006) prior to submission of the IWC reports (Table 1).

#### 3.2.1. Methods within IWC Data

Methods were not reported for 10 (21.7%) stranded cetaceans euthanised in the UK. Chemical methods to euthanise stranded cetaceans were most common in the UK (52.2%, n = 24). The chemical euthanasia agent was reported in all cases and was a barbiturate, with intravenous injection being the most common method (78.3%, n = 18). Chemical euthanasia was not used in NZ.

Ballistics methods were used in 26.1% (n = 12) of cases in the UK. However, in only 42% (n = 5) of cases was the firearm calibre reported, with five different firearms being employed (.243, .308, .22, .270 and shotgun). Of these, four different firearms were used in the euthanasia of one delphinid (blackfish) species, the long-finned pilot whale (*Globicephala melas melas*), with .243 firearm being most common (Table 2). The number of projectiles used was reported in 50% (n = 6) of cases, with a range of 1–3 required (mean = 2, SD = 0.89). However, the projectile characteristics were reported in only 16.7% (n = 2) of cases, with soft-point projectiles reported for a single euthanised cetacean. The approach used (dorso-ventral or lateral) was recorded in only one case, and was described as lateral.

In NZ, only ballistics methods were used between 2007 and 2020, with firearm type (n = 16) recorded in 98% of cases (n = 548). Of these, 10 different firearms were used to euthanise long-finned pilot whales (*Globicephala melas edwardii)*, with a .30-06 firearm being most common (Table 2). However, the projectile characteristics were only recorded for 13% (n = 74) of individuals euthanised; all reported projectiles were soft-point with varying grain from 140 to 180 gr. The number of projectiles required was reported in 68% (n = 379) of cases, ranging from 1 to 6 (mean = 1.3, SD = 0.7). The approach used for firearm discharge was not reported for any individual. Similarly, between 1991 and 2006 ballistics were the only reported method used in NZ, though the method was recorded for only 21 individual euthanised cetaceans (12%). Four different firearm calibres were reported, with no projectile characteristics, and the number of projectiles used varied between 1 and 11 (mean 2.6, SD = 3.1).

#### 3.2.2. Time to Death (TTD) within IWC Data

TTD was not recorded in the UK prior to 2014. In the reported data, 16 (35%) of individual euthanised cetaceans had TTD recorded, with 17.4% (n = 8) presumed instantaneous (Table 1). All cases reported as presumed instantaneous involved ballistics as the method. For those not presumed instantaneous, TTD ranged from 1 to 3 min (mean = 2 min, SD = 30 s) and were related to chemical methods.

In NZ between 2007 and 2020, 84% (n = 472) of animals were reported as instantly killed, with an additional 4% (n = 22) recorded as “presumed instantaneous” (Table 1). Individual cetaceans that were not killed instantly had a reported TTD from 30 s up to 12 h (mean = 55 min, SD = 191 min). TTD data were not recorded for nine individual animals (1.6%). Between 1991 and 2006, only three (2%) individual euthanised cetaceans had TTD recorded, with two (1%) reported as presumed instantaneous.

In the UK, the reported criteria used to assess TTD included “no respiration, no apex beat detectable by palpation or auscultation and no corneal reflex”, however the use of these criteria was only directly reported as used on one animal (2.2%). In NZ, the reports provided a summary of criteria used to assess TTD, including “no further breathing, complete dilation of the pupils; onset of unprovoked agonal convulsions (violent uncoordinated thrashing); absence of palpebral (closure of eyelid when corner of eyelid touched) and corneal (closure of eyelid if eye touched) reflexes and slack lower jaw”. Details of these criteria being implemented following application of euthanasia method were only reported for 0.2% (n = 1) of animals.

#### 3.2.3. Taxa and Stranding Type within IWC Data

In the UK, a total of 46 cetaceans of 10 different species were euthanised between 2014 and 2018. Most (57%, n = 26) were classified as delphinids (Table 1). The stranding type (mass or single) was not provided, except in one case where multiple animals were reported as being euthanised. In NZ, a total of 561 stranded cetaceans of 19 different species were euthanised at stranding events between 2007 and 2020. Most (83%, n = 466) were classified as delphinids (blackfish) (Table 1). Delphinids (blackfish) were also found to dominate the historical DOC data (1991–2006), though a greater proportion of kogiids were reported as euthanised during this earlier time period. Overall, a total of 33 mass and 42 single stranding events were recorded in the NZ data, and a further 30 events were not identified by stranding type.

## 4. Discussion

This study revealed a number of notable gaps in the current reporting of cetacean euthanasia. What was reported suggested that two broad methods are commonly used (i.e., chemical and ballistics), but that the associated approaches and equipment vary. This highlights the need for standardised protocols for the euthanasia of different taxa. Of particular concern was the lack of reporting on the criteria used to assess death and the time from application of the method to confirmed death, which limits our understanding of the duration of any welfare impacts associated with killing.

The low and poorly detailed reporting in much of the peer-reviewed literature regarding the employment of a particular method and the associated TTD likely thwarts any improvements to current practises. Additionally, this lack of data will likely impact the implementation of euthanasia or may result in the practice being carried out inappropriately, resulting in welfare concerns. It is likely that further information exists which may only be discussed during workshops, meetings or in the grey literature (e.g., [40,41]). This may be further exacerbated in some cases by a reluctance to share events that went awry. However, such experiences and information are critical if improvements in euthanasia and related welfare outcomes are to be achieved.

In this study, peer-reviewed articles detailing marine mammals and euthanasia were only found post-1980, which may be because the first Marine Mammal Protection Act (USA) was enacted in 1972, followed by New Zealand in 1978 and the Wildlife and Countryside Act 1981 in the UK, all of which include regulations around the treatment and disposal of sick or injured marine mammals. Following this, a number of workshops were held focussing on humane killing techniques for hunted whales [35] and cetacean stranding events [42]. These workshops may have highlighted research priorities around the killing methods for cetaceans, which then proliferated into published research.

Although reports from the IWC archives provide more data than the peer-reviewed literature, these are limited in the detailed information provided regarding the method and the welfare impact assessments (TTD) undertaken. Furthermore, the UK and NZ reports do not provide insights as to how techniques may be further developed to improve welfare outcomes, despite their submission to a subcommittee of the IWC that focuses on welfare implications.

Currently, the most comprehensive guidance for stranded cetacean euthanasia originate from non-peer-reviewed sources, where the extensive knowledge of experts in the field have been collated [17,43,44,45,46]. Further work should aim to build on this knowledge by improving data collection at euthanasia events. Additionally, where possible, robust scientific trials should be considered to assess methods that will help to strengthen current guidance and welfare outcomes.

### 4.1. Chemical Method

In the UK, chemical methods were the most commonly reported way to kill stranded cetaceans over the four-year reporting period. This is similar to what was found in the literature (91% of articles), where it was noted that chemical euthanasia is often considered as the most reliable and socially acceptable method, likely due to the similarities with companion animal euthanasia [16,18]. Our study also found that the most commonly reported route of administration for chemical euthanasia in the UK was intravenous injection. This was also the case in the data collected through the peer-reviewed literature, where 77% (n = 34) of chemical euthanasia cases involved intravenous injection, with 11 of these describing stranded cetacean euthanasia (e.g., [13,29]). The intra-venous route may be used because it is considered the most rapid and reliable way to humanely euthanise mammals [18], and so has become common practise for marine mammals. However, in moribund cetaceans the peripheral circulation will start to collapse and so the vasculature in the peduncle may be the most accessible site, but this poses danger to personnel working around the flukes during potential excitatory phases. Furthermore, relatively large doses are required due to the large size of cetaceans, which are expensive and the onset of action of the drug may take some time [20]. However, TTD was reportedly fast (1–3 min) following chemical euthanasia in the UK.

Although chemical methods may be more aesthetically pleasing, there are compelling welfare arguments for employing the method that will provide the shortest TTD over public sentiment [13,14]. In this study we found that chemical euthanasia was never reported to cause instantaneous death, with TTD from the peer-reviewed literature varying between 5 min and 49.7 h (mean = 4.7 h, SD = 13 h), and from the UK data ranging from 1 to 3 min (mean = 2 min, SD = 30 s). The delayed TTD is due to the time that it takes to inject the chemical solution and for it to circulate to the heart and brain [20]. Despite the longer TTD during chemical euthanasia, in some cases it will cause less suffering than if inappropriate physical methods are applied or are applied incorrectly. Finally, possible eco-toxicological hazards may occur due to residues bioaccumulating in the environment and there is the possibility of secondary toxicosis [16,47,48,49]—this is one of the primary reasons that such chemical methods are not employed in NZ stranding events (L. Boren DOC, pers. comm.). Though ballistics using lead bullets may also come with their own ecotoxicological risks [50]. Another reason for not employing chemical euthanasia likely relates to the lack of specialist veterinary personnel at stranding events to administer such drugs effectively and safely [17,20].

### 4.2. Ballistics Method

NZ employed only ballistics methods across the 13-year period of reporting to the IWC, with no other methods reported by DOC in the data collected in the 16 years prior, indicating this is likely the only method employed. In the UK, ballistics were also employed on 26.1% of individual cetaceans. However, this method was much less commonly reported in the peer-reviewed literature, with only 12% of articles describing its application. Physical methods such as ballistics are often preferred for the killing of medium-sized mammals as they can be instantaneous, do not require veterinary expertise and pose less contamination risk than chemical methods [17]. Although ballistics have been demonstrated as effective on small cetaceans (<6 m) [23,51], the most effective orientation at discharge (dorso-ventral or lateral) and studies of euthanasia via ballistics for larger cetaceans (>6 m) are lacking. The type of firearm and projectiles used should differ depending on species anatomy and size, with larger animals requiring a higher muzzle energy [18,23] (i.e., high calibre firearms), and large projectiles are necessary for larger cetaceans [22]. The inappropriate discharge of a firearm on a cetacean can cause negative welfare impacts, yet few studies have examined the likelihood of ballistics causing instantaneous death by examining cranial pathology [22,23,51].

In NZ, sixteen different firearm calibres were reported, including the most prevalent being .30 calibre (.30-06, .300, .303, .308) accounting for 89% (n = 504) of cases between 2007 and 2020. The few firearms reported in the UK were similar to those reported in NZ and in the wider literature [23,52]. The wide range of firearms reported by NZ and the UK likely represent the variety that may be employed elsewhere. Such an array of firearm types, calibres and associated projectiles may mean that equipment inappropriate for the euthanasia of cetacean species is employed. This could cause animals to be severely injured but remain alive, significantly reducing their welfare [22,23]. This is supported by the data reported in NZ, where we found wide-ranging TTD from 30 secs to 12 h (mean = 55 min, SD = 191 min). Therefore, the wide range of firearm calibres reported suggests that field-testing of these to assess their suitability for different species and sizes of cetaceans would prove useful. This is particularly highlighted where smaller-calibre firearms (e.g., .22, .243) have been employed, evidenced both in the UK and NZ data, and where they are currently part of guidance in standard operating procedures (SOPs) (e.g., [40]). In contrast, recommendations based on ballistics trials on cetacean cadavers have stated that only larger .30 calibre should be employed [23].

The projectile characteristics are as important as the firearm calibre employed for influencing terminal ballistics [53], yet the reported data from the UK and NZ show that projectile characteristics were reported in only 16.7% and 13% of individual cetacean euthanasia cases, respectively. Those reported showed that soft-pointed profile (expanding) projectiles of varying grain were used. Such soft projectiles are also recommended in the NZ SOP [44] for stranded cetacean euthanasia, though no detail on their required profile is provided. Another SOP [45] for Western Australia, which based its recommendations on ballistics testing [23], states that only solid projectiles should be used. Furthermore, a clinical report by NZ veterinarians also recommends the use of only “rifle of calibre 0.303 or greater and solid bullets” for all stranded cetaceans [54]. Such recommendations are due to the fact that soft-point bullets have proven unreliable due to lower penetration depth [45,55] and lack killing efficiency [18,22,56].This is due to the unique cranial anatomy of cetaceans, where the skin, thick blubber and muscle around the cetacean melon absorb kinetic energy. Furthermore, the anterior surface of the thick cranium is also concave with extensive sinuses which are likely to cause bullet deflection [42]. This means that non-expanding projectiles (solid) should be used to ensure maximum penetration depth with minimum projectile deviation [18,43]. The reasons why NZ is using and recommending the use of soft-point projectiles is unknown, though it may simply be due to projectile availability.

To ensure that euthanasia via ballistics is humane, the brain should be destroyed instantly [57]. Typically, this is achieved by aiming for the occipital condyles in order to cause instantaneous death [8,51]. There are two main orientations for this target when discharging a firearm: dorso-ventral and lateral. These orientations were tested in a ballistics trial in NZ which found that dorso-ventral was most appropriate for smaller cetaceans and lateral for larger cetaceans [51]. Despite this study being well cited in other publications [23] and guidelines (e.g., [40,42]), the orientation employed for euthanasia was rarely reported. In this study we found four peer-reviewed articles, all of which reported the use of dorso-ventral orientation and two of lateral orientation. Similarly, in the data reported to the IWC, only the UK provided the orientation applied for the euthanasia of a single stranded cetacean. The orientation of discharge of a firearm will be affected by the positioning of the stranded cetacean and the species involved. It has previously been noted that the extensive muscle on the nuchal, parietal and occipital regions of the cetacean skull mean that occipital shooting will be ineffective [42]. Furthermore, the unique cranium anatomy of cetaceans also varies between species. Therefore, it is important that the orientation of discharge is appropriate as suggested in guidelines [17,45] and is also reported, as this will provide species-specific knowledge regarding the most appropriate orientation and external anatomical landmarks to ensure correct shot placement and instantaneous death [17,23].

### 4.3. Taxa Euthanised

In the peer-reviewed literature that examined the euthanasia of wider marine mammal taxa, pinnipeds were most commonly reported on. However, when looking specifically at those articles that described euthanasia related to stranding events (n = 44), delphinids (including blackfish) and mysticetes were the subjects of most articles. Similarly, the euthanasia data reported to the IWC were focussed on delphinids. In the UK, the euthanasia of delphinids was most commonly reported, whilst in NZ the cetacean taxa most commonly reported as euthanised was delphinid (blackfish). The majority of these individuals were pilot whales, which primarily reflects their high stranding incidence [58]. However, it may also relate to the fact that smaller cetaceans such as delphinids are considered to be simpler to humanely kill, in comparison to larger cetaceans such as mysticetes. This highlights the global need to increase ballistics studies and knowledge on how to humanely kill larger cetacean species. The data reported to the IWC also highlight the fact that there are a wide range of species (n = 23) reported as stranded and euthanised in NZ and the UK. This further supports the notion that additional work on euthanasia methods is required in order to ensure that the most appropriate method and associated equipment are used for the species in question. In terms of ballistics, this should include ballistics trials on cadavers to ensure the most appropriate approach, firearm and projectile are employed, particularly in relation to the varying skull morphology between species [59]. For chemical euthanasia, this should include detailed documentation of the chemical agent and associated sedatives used, along with details of the needle gauge, dosages and any behavioural reaction that may occur [17].

Likely due to the layout of the IWC reporting forms, the type of stranding event (mass or single) was generally not recorded in the reviewed reports. However, euthanasia at mass stranding events is likely to be more complex to manage due to the number of animals and often the variety of stakeholder views, which may make end of life decisions particularly contentious [60,61]. It has also previously been suggested that exposure of animals to the noise and visual destruction of their conspecifics may increase their anxiety and fear [62], suggesting a possible reduction in the welfare of conscious mass-stranded cetaceans during the euthanasia of their moribund pod members. This highlights the need to euthanise multiple individuals rapidly, but this may also mean that carrying out individual assessments of TTD becomes logistically difficult, as was stated in one NZ report as the reason TTD data was not collected for each individual [63]. This is despite the fact that such data is imperative to assess welfare impacts and ensure humane killing.

### 4.4. Time to Death (TTD)

TTD was rarely reported in the literature, with only 10 (0.5%) articles reporting TTD data. In the reports to the IWC, the UK provided TTD for 35% of individuals, but it was also noted in one of the reports that such TTD data was only starting to be collected after 2014. NZ, on the other hand, reported TTD for almost all individuals (98%) between 2007 and 2020—a notable improvement in reporting when compared to the 1991–2006 data. However, a number of the NZ cases were reported as “presumed instantaneous”, highlighting uncertainty as to how death was being confirmed. Unsurprisingly, our study found that reported instantaneous death only occurred when employing physical euthanasia methods, such as ballistics and explosives. This is because chemical euthanasia takes time from the point of injection for the agent to circulate to the heart and brain [20]. Although death from chemical euthanasia was not instant, in the reported IWC data it was not vastly variable (1–3 min). This suggests lower welfare impacts at the population level from chemical euthanasia reported in the IWC data compared with death by ballistics, which varied widely from 30 secs to 12 h.

For most individuals in the UK and NZ, details on the criteria used to assess death were not provided. The verification of death is imperative when the euthanasia of an animal is carried out [64]. Because the assessment of death using “gold standards” such as cessation of cardiac activity [26] can be complex in cetaceans, the implementation of multiple criteria should be used to confirm death or at least insensibility [27,28]. Although the NZ SOP requires the verification of death and provides details on the criteria used to assess death following Knudsen (2005), one report [63] examined actually stated that “TTDs were not recorded for individual whale at (……) but were estimated to all be under 3 min”. No details were provided as to why 3 min was the estimation, and a further nine animals had no TTD recorded. This reported lack of assessment of death or insensibility in these euthanised cetaceans and the unverified assumption of death leaves significant uncertainty regarding the welfare impacts of the killing.

The difficulties in assessing, and the lack of validation of, the criteria for death or insensibility in cetaceans also limits our current understanding of the humaneness of methods. Although current guidelines recommended by the IWC and followed in SOPs [44,45] are those suggested by [24], these differ from the criteria suggested as reliable for assessing insensibility and death collated through expert opinion [27,28]. These criteria are more similar to those reported in the peer-reviewed literature, including cessation of cardiac movements and loss of palpebral and corneal reflexes. The current criteria recommended by the IWC were originally developed for use in the humane killing of cetaceans hunted at sea, however this has limited the assessment of their validity due to logistical complexities. The implementation of all recommended criteria [28] and the examination of other criteria not yet implemented at stranding events could greatly enhance our understanding of the humaneness of killing procedures. However, there is a need to assess the validity of all recommended criteria as has been done for domesticated animals [65,66].

## 5. Conclusions

Historically, few peer-reviewed articles have focussed on the topic of marine mammal euthanasia, and those that have mentioned euthanasia have provided little detail on how killing was achieved and how long it took for animals to die. Greater detail has been reported to the IWC for stranded cetacean euthanasia by the UK and NZ in recent years. The available data suggest that chemical and ballistics methods are most commonly employed, with some geographical differences, but that detailed reporting of equipment is lacking. They also highlight that most euthanasia events involve delphinids, which may be in part due to their high incidence of stranding, but is also likely due to the increased complexities inherent in euthanising larger and unusual species. The data from IWC also lack some important information, such as detail on the projectile characteristics and orientation of discharge used for ballistics. Notably, little information is reported on the criteria for death that were assessed for each individual, reducing the ability to assess the welfare impacts of killing. Furthermore, just 2 of the 88 member nations of the IWC have reported on stranded cetacean euthanasia, highlighting how a simple increase in the reporting rate could significantly improve our knowledge of methods and welfare impacts globally.

Not only is further work on methods of killing required to assess humaneness, but validation of criteria used for assessing death or insensibility is needed to enhance our understanding of the welfare impacts of killing methods. The assessment and detailed reporting of the species, method, TTD and time to insensibility following euthanasia of an individual could improve our understanding of the welfare impacts from particular techniques and provide species-specific guidance. This improved knowledge would also allow managers to educate the wider community on the importance of euthanasia and appropriate methods as a viable welfare-oriented option for stranded cetaceans with low survival likelihood. Overall, such improvements would result in the best welfare outcomes for compromised stranded cetaceans.

## Figures and Tables

**Table 1:** Data collated from International Whaling Commission (IWC) reports and historical records of individual stranded cetacean euthanasia from the United Kingdom (UK) and New Zealand (NZ). NA = not applicable.

		UK	NZ	NZ
	Years of data	2014–2018	2007–2020	1991–2006
	Total no. individuals euthanised	46	561	180
	No. species euthanised	10	19	13
	Method not reported	21.7% (n = 10)	0% (n = 0)	88% (n = 159)
Chemical methods	% of individuals chemically euthanised	52.2% (n = 24)	NA	NA
	Chemical agent reported	100% (n = 24)	NA	NA
	Types of injection routes reported Intra-venous (IV) Intra-cardiac (IC) Intra-muscular (IM) Intra-thoracic (IT) Intra-peritoneal (IP)	78.3% (n = 18) 8.7% (n = 2) 4.3% (n = 1) 4.3% (n = 1) 4.3% (n = 1)	NA	NA
Ballistics methods	% of individuals ballistically euthanised	26.1% (n = 12)	100% (n = 561)	12% (n = 21)
	Firearm reported	42% (n = 5)	98% (n = 548)	43% (n = 9)
	No. firearm calibres reported	5	16	4
	No. projectiles reported	50% (n = 6); range: 1–3 (mean = 2, SD = 0.89)	68% (n = 379); range: 1–6 (mean = 1.3, SD = 0.7)	43% (n = 9); range: 1–11 (mean = 2.6, SD = 3.1)
	Projectile characteristics reported	16.7% (n = 2)	13% (n = 74)	0
	Orientation reported	n = 2: lateral	0	0
Assessment of death/insensibility	TTD reported	35% (n = 16)	98.4% (n = 552)	2% (n = 3)
	Presumed instantaneous death reported	17.4% (n = 8)	4% (n = 22)	1% (n = 2)
	Instantaneous death reported	0	84% (n = 472)	0
	TTD from all methods employed	range: 1–3 min (mean = 2 min, SD = 30 secs)	Range: 30 secs–12 h (mean = 55 min, SD = 191 min)	Range: 0–5 min (mean = 5, SD = 0)
	Criteria to assess death reported	2.2% (n = 1)	0.2% (n = 1)	0
Taxa	Mysticete	2.2% (n = 1)	1.6% (n = 9)	4% (n = 7)
	Ziphiid	2.2% (n = 1)	2.1% (n = 12)	4% (n = 7)
	Delphinid	57% (n = 26)	2.9% (n = 16)	8.3% (n = 15)
	Delphinid (blackfish)	19.5% (n = 9)	83% (n = 466)	64.4% (n = 116)
	Kogiid	N/A	10% (n = 58)	19.4% (n = 35)
	Phocoenid	20% (n = 9)	N/A	N/A

**Table 2:** Number of reported individual cetaceans euthanised using ballistics per taxon and per firearm type in United Kingdom (UK) and New Zealand (NZ) based on available International Whaling Commission (IWC) data between 2007 and 2020.

	NZ					UK					
Firearm Calibre	Mysticete	Ziphiid	Delphinid	Kogiid	Delphinid (Blackfish)	Mysticete	Ziphiid	Delphinid	Delphinid (Blackfish)	Phocoenid	Total Individuals Euthanised per Firearm Type
.22			2							1	3
.223			2								2
.243			2		1				2		5
.270	2			1	19				1		23
.300				1							1
.303	1	4	1	18	151						175
.308	2	3	3	11	58				1		78
.30-06	3	2	1	27	219						252
.357			1								1
.416	1										1
.44 magnum		2									2
Bolt-action rifle 7mm-08					1						1
Boltgun			1								1
Bushmaster semiauto 7.62x39SP					6						6
Rifle 6.5x55					7						7
Shotgun			2		1				1		4
Unknown		1	1		3	1	1	1	3		11
Total individuals euthanised using ballistics per taxon	9	12	16	58	466	1	1	1	8	1	573

## Data Availability

Publicly available datasets were analysed in this study. These data can be found here: https://archive.iwc.int/pages/home.php?login=true. Restrictions apply to the availability of these data. Data were obtained from Department of Conservation New Zealand and are available from the Department of Conservation Head Office, Wellington, New Zealand with the permission of the Department of Conservation New Zealand.

## Supplementary Materials

The following are available online at https://www.mdpi.com/article/10.3390/ani11051460/s1. Table S1: Marine mammal euthanasia via chemical methods (injection and inhalation), including details of methods and time to death (TTD) as reported in the peer-reviewed literature between 1980 and 2020. Table S2. Marine mammal euthanasia via physical methods (ballistics and explosives), including details of methods and time to death (TTD) as reported in the peer-reviewed literature between 1980 and 2020. Table S3. Cetacean species reported as euthanised using chemical methods and respective chemical information and time to death (TTD) as reported by the United Kingdom (UK) to the International Whaling Commission (IWC) between 2014 and 2018. Table S4. Cetacean species reported as euthanised using ballistics methods and respective ballistics information and time to death (TTD) as reported by the United Kingdom (UK) to the International Whaling Commission (IWC) between 2014 and 2018. Table S5. Cetacean species reported as euthanised using ballistics methods and respective ballistics information and time to death (TTD) as reported by New Zealand (NZ) to the International Whaling Commission (IWC) between 2007 and 2020.
