# Supplementary material for: Deathly Silent: Exploring the Global Lack of Data Relating to Stranded Cetacean Euthanasia
Source: Animals (Basel). 2021 May 19;11(5):1460. doi: 10.3390/ani11051460 (PMC8161157; doi:10.3390/ani11051460)
Supplement: Supplementary file 1 [file animals-11-01460-s001.zip › animals-1185152-supplementary.pdf]

Table S1. Marine mammal euthanasia via chemical methods (injection and inhalation), including details of methods and time to death (TTD) as reported in the peer-reviewed literature between 1980 and 2020.

Taxon	Species	Method	Length of animal (m)	Mass of animal (kg)	Chemical agent	Parenteral injection route	Injection location	Dose	TTD (mins)	Ref.
Cetacean	Cetacean	Chemical			Barbiturate					[14]
Delphinid	Bottlenose dolphin	Chemical			Barbiturates	IV				[3]
Delphinid	Common dolphin	Chemical	1.91		Pentobarbitone Euthatol	IP	Peritoneal cavity	60 mL	20	[15]
Delphinid	Common dolphin	Chemical			Barbiturates	IV				[3]
Delphinid	White-beaked dolphin	Chemical	2.66	273.7	Midazolam	IM		0.08 mg/kg	20	[59]
					Acepromazine	IM		0.8 mg/kg		
					Xylazine	IM		1.6 mg/kg		
Delphinid (Blackfish)	False Killer whale	Ballistics, Chemical			Pentobarbitone					[16]
Delphinid (Blackfish)	Long-finned pilot whale	Chemical			T61	IV	Dorsal fin	0.14 cc per pound of body weight		[38]
Delphinid (Blackfish)	Long-finned pilot whale	Chemical			Pentobarbital					[39]
Delphinid (Blackfish)	Long-finned pilot whale	Chemical	5.54, 5.55		Etorphine			2.45 mg/mL		[40]
					Acepromazine			10 mg/mL		
Delphinid (Blackfish)	Melon-headed whale	Chemical	2.12, 2.40, 2.48	125, 200, 125	Xylazine	IM	Epaxial muscles	0.5 mg/kg	8	[41]
					Ketamine	IM	Epaxial muscles	2.5 mg/kg		
Delphinid (Blackfish)	Pilot whale	Chemical	2.1		Diazepam	IM		20 mg		[43]
					Sodium pentobarbital	IH		23,400 mg		
					Phenytoin	IH		300 mg		
Delphinid (Blackfish)	Pilot whale	Chemical	2.97		Meperidine	IM		100 mg		[43]

					Sodium pentobarbital	IC	19,500 mg	
					Phenytoin	IC	2500 mg	
Delphinid (Blackfish)	Pilot whale	Chemical	3.58		Xylazine	IM	6000 mg	[43]
					Acepromazine	IM	100 mg	
Delphinid (Blackfish)	Pilot whale	Chemical	3.52		Sodium pentobarbital		46,800 mg	[43]
					Phenytoin		6000 mg	
Delphinid (Blackfish)	Pilot whale	Chemical	3.59		Xylazine	IM	1000 mg	[43]
					Acepromazine	IM	100 mg	
					Sodium pentobarbital		46,800 mg	
					Phenytoin		6000 mg	
Delphinid (Blackfish)	Pilot whale	Chemical	3.5		Sodium pentobarbital		46,800 mg	[43]
					Phenytoin		6000 mg	
Delphinid (Blackfish)	Pilot whale	Chemical	3.49		Xylazine	IM	6000 mg	[43]
					Acepromazine	IM	100 mg	
Delphinid (Blackfish)	Pygmy killer whale	Chemical	2.46		T61	IV		[53]
Kogiid	Pygmy sperm whale	Chemical	3.1		Midazolam	IM	50 mg (5 mg/mL)	[54]
					Pentobarbital (Euthasol)	IV		
Mustelid	Southern sea otter	Chemical	0.45	1.45	Fentanyl citrate, Diazepam	IM		[56]
					Pentobarbital sodium	IV		
Mustelid	Southern sea otter	Chemical			Pentobarbital	IV	2340 mg	[57]
					Pentobarbital	IV	5850 mg	

Mysticete	Fin whale	Chemical	10.5		Xylazine	IV	Major vein close to heart/thorax	5000 mg	60	[17]
					T61	IV	Major vein close to heart/thorax	100 mL		
					KCl	IV	Major vein close to heart/thorax	1200 mmol/600 mL sterile water		
Mysticete	Fin whale	Chemical, Inhalation	13.5		Pentobarbital, T61	Inhalation	Blowhole	60 mL (390 mg/mL)	40	[18]
					T61	IV	Fluke	2 x 60mL		
Mysticete	Gray whale	Chemical	8.22		Pentobarbital	IV	Ventral margin of peduncle	500 cc		[19]
Mysticete	Gray whale	Chemical	8–12		Midazolam	IM		0.02 mg/kg		[20]
					Pentothal sodium	IV	Superficial caudal peduncle veins			
Mysticete	Humpback whale	Ballistics, Chemical			Detomidine, Midazolam, Butorphanol	IM	12-inch dart			[36]
					Barbiturate	IV, IP				
Mysticete	Humpback whale	Chemical	8.78	9500	Midazolam	IM	Left epaxial muscle cranial to scapula	400 mg (0.04 mg/kg)	99	[37]
					Acepromazine	IT	Caudal to right pectoral fin	1950 mg (0.2 mg/kg)		
					Xylazine	IV	Right pectoral fin between ulna and radius	31,000 mg (3.4 mg/kg)		

					KCl	IC, IV	Near heart	100 mg/kg (1.3 mmol/kg)		
Mysticete	Humpback whale	Chemical	8.3	8000	Midazolam	IM		415 mg (0.05 mg/kg)	138	[37]
					Acepromazine	IM		2100 mg (0.25 mg/kg)		
					Xylazine	IM, Retrobulbar, IV		30,000 mg (3.5 mg/kg)		
					KCl	IT, IV		220 mg/kg (3 mmol/kg)		
Mysticete	Humpback whale	Chemical	9.76	13,000	Midazolam	IM		325 mg (0.025 mg/kg)	109	[37]
					Acepromazine	IM		2000 mg (0.15 mg/kg)		
					Xylazine	IV	Right pectoral fin vessel	33,500 mg (2.6 mg/kg)		
					KCl		Near heart	150 mg/kg (2 mmol/kg)		
Mysticete	Minke whale	Chemical	3.04	276	Midazolam	IM		0.14 mg/kg	48	[37]
					Acepromazine	IM, IV		1.6 mg/kg		
					Xylazine	IM, IV, IC		15.3 mg/kg		
					KCl	IC		168 mg/kg (2.2 mmol/kg)		
Mysticete	Minke whale	Chemical	5.8		Barbiturates	IV				[42]
Mysticete	Minke whale	Chemical	2.84		Diazepam	IM		20 mg		[43]
					Sodium pentobarbital	IH		70,200 mg		
					Phenytoin	IH		9000 mg		
Mysticete	Minke whale	Chemical	2.93	214.8	Meperidine (Meperidine HCl)	IM		100 mg (0.46 mg/kg), (25 mg/mL)	31	[44]
					Pentobarbital sodium	IC		13.65 g (63 mg/kg)		

					Phenytoin sodium	IC		1.75 g (8.1 mg/kg)		
Mysticete	Right whale	Chemical, Inhalation, Exsanguination	9.75	10,000	Midazolam	Retrobulbar		90 mg (0.009 mg/kg)	123	[37]
					Diazepam	Retrobulbar		150 mg (0.015 mg/kg)		
					Acepromazine	Retrobulbar		450 mg (0.045 mg/kg)		
					Xylazine	Retrobulbar		13,000 mg (1.3 mg/kg)		
					Medetomidine	Retrobulbar		22 mg (2.2 µg/kg)		
					Isoflurane	Inhalation		200 mL		
Mysticete	Southern Right whale	Chemical	14	40,000	Xylazine	IM	Tongue	8000 mg (0.2 mg/kg), 19,800 mg (0.5 mg/kg)	2981	[55]
					Midazolam	IM	Tongue	840 mg (0.021 mg/kg)		
					Thiopental sodium in saline	Inhalation	Blowhole	113 g in 2000 mL		
					Chloral hydrate	Inhalation	Blowhole	500 g		
					Ketamine	IM	Tongue	99,000 mg (2.47 mg/kg)		
					T61	IV	Near heart	750 mL		
					KCl	IV	Near heart	10,000 mL of 1 mmol		
Odontocete	Sperm whale	Chemical	6.1		Barbiturate Beuthanasia-D Special	IV	Left pectoral fin	20 cc	5	[58]
					Barbiturate Beuthanasia-D Special	IV	Vein on dorsal side of fluke	20 cc		

Pinniped	Antarctic fur seals	Chemical			Tiletamine hydrochloride, Zolazepam hydrochloride			1:1	[1]
Pinniped	Bearded seal	Chemical	1.8	92.8	Telazol	IV	Epidural vein		[2]
Pinniped	California sea lion	Chemical			Xylazine hydrochloride<br>Sodium pentobarbital	IM<br>IC		2.5 mL of 100 mg/mL<br>40 mL of 389 mg/mL	[10]
Pinniped	California sea lion	Chemical			Tiletamine Zolazepam (Telazol)<br>Pentobarbital	IM<br>IV, IC		175 mg 1:1, 100 mg<br>2730 mg	[11]
Pinniped	California sea lion	Chemical			Pentobarbital	IV			[12]
Pinniped	California sea lion	Chemical		37	Tiletamine-zolazepam<br>Pentobarbital, Phenytoin sodium	IM<br>IV	 Subclavian vein	200 mg<br>10 mL of 39% and 5%	[4]
Pinniped	California sea lion	Chemical, Inhalation			Telazol<br>Metatomadine<br>Isoflurane	IM<br>IM<br>Inhalation		1.0 mg/kg<br>0.04 mg/kg	[5]
Pinniped	California sea lion	Chemical			Barbiturate				[50]
Pinniped	California sea lion	Chemical			Pentobarbital sodium, Phenytoin sodium	IV	Subclavian vein	1 mL/5 kg of 39% and 5%	[6]
Pinniped	California sea lion	Chemical, Inhalation							[60]
Pinniped	California sea lion	Chemical		13–168	Pentobarbital	IV	Subclavian vein	80 mg/kg	[7]
Pinniped	California sea lion	Chemical			Pentobarbital sodium,	IV	Subclavian vein	1 mL/5 kg of 39% and 5%	[8]

					Phenytoin sodium				
Pinniped	California sea lion	Chemical			Barbiturate				[9]
Pinniped	Cape fur seal	Chemical	1.93	182	Sernylan	IM		500 mg	[13]
					Rompun	IM		60 mg	
					Scoline	IM		100 mg	
Pinniped	Grey seal	Chemical		58.5	Euthanyl				[21]
Pinniped	Grey seal	Chemical		155	Pentobarbital sodium (Euthasol)	IV		70 mL	[22]
Pinniped	Grey seal	Chemical		19	Pentobarbitone (Euthatal)	IV	Extradural intravertebral vein	150 mg/kg	[23]
Pinniped	Grey seal	Chemical			Pentobarbital	IV			[24]
Pinniped	Grey seals	Chemical			T61			0.3 mL/kg	[31]
Pinniped	Harbor seal	Chemical			Pentobarbitone	IV		1900 mg	[25]
Pinniped	Harbor seal	Chemical		90					[26]
Pinniped	Harbor seal	Chemical			Pentobarbital sodium, Phenytoin sodium	IV		5 mL of 39% and 5%	[27]
Pinniped	Harbor seal	Chemical, Inhalation							[60]
Pinniped	Harbor seals	Chemical			Sodium pentobarbital	IV		0.5 mL/kg	[28]
Pinniped	Harbor seals	Chemical			T61			0.3 mL/kg	[29]
Pinniped	Harbor seals	Chemical		120	Pentobarbital sodium, Phenytoin sodium	IV		10,140 mg	[30]
				70	Pentobarbital sodium, Phenytoin sodium	IV		2340 mg	

				57	Pentobarbital sodium, Phenytoin sodium	IV		8800 mg	
				90.3	Pentobarbital sodium, Phenytoin sodium	IV		5460 mg	
Pinniped	Harbor seals	Chemical			T61			0.3 mL/kg	[31]
Pinniped	Harp seal	Chemical			Pentobarbital	IV	Extradural intravertebral vein	15 mL (240 mg/mL)	[32]
Pinniped	Harp seal	Ballistics, Chemical, Exsanguination			Barbiturate	IV		30 mg/kg	[33]
Pinniped	Harp seal	Chemical, Exsanguination			Barbiturate	IV		30 mg/kg	[34]
Pinniped	Hooded seal	Ballistics, Chemical, Exsanguination			Barbiturate	IV		30 mg/kg	[33]
Pinniped	Hooded seal	Chemical, Exsanguination			Barbiturate	IV		30 mg/kg	[34]
Pinniped	Hooded seal	Chemical, Exsanguination			Pentobarbital	IV	Extradural intravertebral vein	20 mg/kg	[35]
Pinniped	New Zealand fur seal	Chemical	1.56	47.5	Barbiturates	IC			[45]
Pinniped	Northern elephant seal	Chemical		42.5	Pentobarbital, Phenytoin sodium	IV	Epidural venous sinus	10 mL of 39% and 5%	[4]
Pinniped	Northern elephant seal	Chemical		60	Pentobarbitone	IV	Extradural intravertebral sinus	20 mL	[46]
Pinniped	Northern elephant seal	Chemical			Pentobarbitone	IV	Extradural intravertebral sinus	20 mL	[47]
Pinniped	Northern elephant seal	Chemical		37.5	Pentobarbital	IV		3.9 g	[48]

Pinniped	Northern elephant seal	Chemical		196	Pentobarbital Beuthanasia	IV	Epidural sinus		[49]
Pinniped	Northern elephant seal	Chemical			Barbiturate				[50]
Pinniped	Northern elephant seal	Chemical			Pentobarbital	IV		389 mg/mL	[51]
Pinniped	Northern elephant seal	Chemical, Inhalation							[60]
Pinniped	Pacific Harbor seal	Chemical			Pentobarbital	IV		389 mg/mL	[51]
Pinniped	Pinnipeds	Chemical			Barbiturate				[14]
Ursid	Polar bear	Chemical			Pentobarbital				[52]

Table S2. Marine mammal euthanasia via physical methods (ballistics and explosives), including details of methods and time to death (TTD) as reported in the peer-reviewed literature between 1980 and 2020.

Taxon	Species	Method	Length of animal (m)	Mass of animal (kg)	Firearm	Projectile characteristics	Orientation	Explosives	TTD (mins)	Ref
Delphinid	Bottlenose dolphin	Ballistics	1.3–2.7		0.300	180 g blunt non-deforming solid	Dorso-ventral			[66]
Delphinid	Common dolphin	Ballistics	2.5–5		12 gauge shotgun	28 g slug, buckshot	Lateral, Dorso-ventral			[61]
Delphinid	Common dolphin	Ballistics								[62]
Delphinid	Common dolphin	Ballistics	1.8		0.308	180 g blunt non-deforming solid	Dorso-ventral			[66]
Delphinid (Blackfish)	False Killer whale	Ballistics, Chemical								[16]
Delphinid (Blackfish)	Long-finned pilot whale	Ballistics	2.5–5		12 gauge shotgun	28 g slug, buckshot	Lateral, Dorso-ventral			[61]
Delphinid (Blackfish)	Long-finned pilot whale	Ballistics								[64]
Delphinid (Blackfish)	Long-finned pilot whale	Ballistics			.30-06		Lateral, Dorso-ventral			[65]
Delphinid (Blackfish)	Risso's dolphin	Ballistics	2.7		0.308	180 g blunt non-deforming solid	Dorso-ventral			[66]

Kogiid	Pygmy sperm whale	Ballistics	1.8–2.5		.300, .308	180 g blunt non-deforming solid	Dorso-ventral			[66]
Mysticete	Humpback whale	Ballistics, Chemical			0.577	3 rounds	Pericranial			[36]
Mysticete	Humpback whale	Explosives	9.11	10000			Dorso-ventral	6 x AN60	0	[63]
			10.5	15000	0.300	5 rounds	Dorso-ventral	5 X 125g Powergel magnum		
			9.8	15000				14 X 125g Powergel magnum	0	
			12.7					22X 125g Powergel magnum and 2X 50g Boosters	0	
			9.5	15000				15X 125g Powergel magnum	0	
Mysticete	Humpback whale	Ballistics	4.2–4.3		0.300	180 g blunt non-deforming solid	Dorso-ventral			[66]
Pinniped	Harp seal	Ballistics, Chemical, Exsanguination								[33]
Pinniped	Hooded seal	Ballistics, Chemical, Exsanguination								[33]
Ziphiid	Cuvier's beaked whale	Ballistics	4.2		0.300	180 g blunt non-deforming solid	Dorso-ventral			[66]

Table S3. Cetacean species reported as euthanised using chemical methods and respective chemical information and time to death (TTD) as reported by the United Kingdom (UK) to the International Whaling Commission (IWC) between 2014 and 2018.

Taxon group	Species	Number individuals euthanised	Chemical agent	Injection route	Dose reported	TTD (mins)
Delphinid	Atlantic white-sided dolphin	1	Pentobarbitone	Intravenous	100 mL	
Delphinid	Bottlenose dolphin	1	Somulose	Intravenous	50 mL	
Delphinid	Bottlenose dolphin	1	Pentoject	Intravenous	3 x 50 mL	<2

Delphinid	Common dolphin	1	Pentobarbital	Intravenous	40 mL at 200 mg/mL	
Delphinid	Common dolphin	1	Pentobarbitone	Intravenous	30 mL	
Delphinid	Common dolphin	1	Pentobarbitone 20%	Intravenous	30 mL	2
Delphinid	Common dolphin	1	Euthatal	Intravenous	50 mL	<1
Delphinid	Common dolphin	1	Pentobarbital	Intravenous	50 mL	<2
Delphinid	Common dolphin	1	Euthatal	Intravenous	50 mL	
Delphinid	Common dolphin	1	Pentobarbitone	Intravenous	50 mL	
Delphinid	Striped dolphin	1	Pentobarbitone	Intravenous	50 mL	2
Delphinid	Striped dolphin	1	Barbiturate	Not provided	Not provided	
Delphinid	Striped dolphin	1	Pentobarbiturate	Intrathoracic	30 mL	
Delphinid	White-beaked dolphin	1	Pentobarbitone	Intravenous	100 mL	
Delphinid	White-beaked dolphin	1	Pentobarbital sodium	Intravenous	100 mL	<3
Delphinid (Blackfish)	Risso's dolphin	1	Pentobarbitone	Intramuscular	100 mL	
Phocoenid	Harbour porpoise	1	Euthatal	Intravenous	20 mL	
Phocoenid	Harbour porpoise	1	Pentobarbital	Intravenous	25 mL	
Phocoenid	Harbour porpoise	1	Barbiturate	Intravenous	20 mL	
Phocoenid	Harbour porpoise	1	Dolethal	Intracardiac	40 mL	
Phocoenid	Harbour porpoise	1	Euthatal	Intraperitoneal	20 mL	
Phocoenid	Harbour porpoise	1	Dolethal	Intravenous	15 mL	<2
Phocoenid	Harbour porpoise	1	Pentobarbiturate, Sedation: medetomidine, ketamine	Intracardiac	20 mL	
Phocoenid	Harbour porpoise	1	Pentoject	Intravenous	25 mL	2

Table S4. Cetacean species reported as euthanised using ballistics methods and respective ballistics information and time to death (TTD) as reported by the United Kingdom (UK) to the International Whaling Commission (IWC) between 2014 and 2018.

**Taxon group**	**Species**	**Number individuals euthanised**	**Firearm**	**Number shots**	**Projectile characteristic**	**Orientation discharge**	**TTD (mins)**
Delphinid	White-beaked dolphin	1	Unknown				

Delphinid (Blackfish)	Long-finned pilot whale	Multiple	.243	1	100 grain	Lateral	
Delphinid (Blackfish)	Long-finned pilot whale	1	Unknown				Presumed instant
Delphinid (Blackfish)	Long-finned pilot whale	1	Unknown				Presumed instant
Delphinid (Blackfish)	Long-finned pilot whale	1	Unknown				Presumed instant
Delphinid (Blackfish)	Long-finned pilot whale	1	.308	3		Lateral	Presumed instant
Delphinid (Blackfish)	Long-finned pilot whale	1	Shotgun				
Delphinid (Blackfish)	Long-finned pilot whale	1	.270		Soft point		
Delphinid (Blackfish)	Risso's dolphin	1	.243	2			Presumed instant
Mysticete	Minke whale	1	Unknown	3			Presumed instant
Phocoenid	Harbour porpoise	1	.22	1			Presumed instant
Ziphiid	Cuvier's beaked whale	1	Unknown	2			Presumed instant

Table S5. Cetacean species reported as euthanised using ballistics methods and respective ballistics information and time to death (TTD) as reported by New Zealand (NZ) to the International Whaling Commission (IWC) between 2007 and 2020.

**Taxon group**	**Species**	**Number individuals euthanised**	**Firearm**	**Number shots**	**Projectile design**	**Projectile grain**	**Number reported as TTD instant**	**Number presumed instant**	**TTD (mins)**
Delphinid	Bottlenose dolphin	1	.357	1			1		
Delphinid	Bottlenose dolphin	1	Shotgun	2			1		
Delphinid	Bottlenose dolphin	1	Shotgun	1			1		
Delphinid	Bottlenose dolphin	1	.243	1			1		
Delphinid	Common dolphin	1	.22				1		

Delphinid	Common dolphin	1	.223	2				1
Delphinid	Common dolphin	1	.308	1				0.3
Delphinid	Common dolphin	1	.308	1			1	
Delphinid	Common dolphin	1	.308	1			1	
Delphinid	Common dolphin	1	.303	1			1	
Delphinid	Dusky dolphin	1	.223	3			1	
Delphinid	Dusky dolphin	1	.22	2			1	
Delphinid	Dusky dolphin	1	Unknown					
Delphinid	Hector's dolphin	1	Boltgun				1	
Delphinid	Southern Right whale dolphin	1	.30-06					
Delphinid	Striped dolphin	1	.243	1			1	
Delphinid (Blackfish)	Long-finned pilot whale	48	.30-06	1			48	
Delphinid (Blackfish)	Long-finned pilot whale	48	.303	1			48	
Delphinid (Blackfish)	Long-finned pilot whale	1	.30-06	2	soft point	150 gm	1	
Delphinid (Blackfish)	Long-finned pilot whale	32	.30-06	1	soft point	150 gm	32	
Delphinid (Blackfish)	Long-finned pilot whale	2	.30-06	2	soft point	150 gm		2
Delphinid (Blackfish)	Long-finned pilot whale	27	.30-06	1	soft point	150 gm	27	
Delphinid (Blackfish)	Long-finned pilot whale	1	.30-06	2	soft point	150 gm	1	
Delphinid (Blackfish)	Long-finned pilot whale	1	.243				1	
Delphinid (Blackfish)	Long-finned pilot whale	29	.30-06				28	0.5
Delphinid (Blackfish)	Long-finned pilot whale	6	Bushmaster Semiauto 7.62x39SP	3			6	0.5

Delphinid (Blackfish)	Long-finned pilot whale	1	Bolt-action rifle 7mm-08	5	1		0.5
Delphinid (Blackfish)	Long-finned pilot whale	4	.303				4
Delphinid (Blackfish)	Long-finned pilot whale	43	.308	3	35		4
Delphinid (Blackfish)	Long-finned pilot whale	11	.30-06	2	10		3
Delphinid (Blackfish)	Long-finned pilot whale	8	.303		8		
Delphinid (Blackfish)	Long-finned pilot whale	19	.270	1-3	2		<2
Delphinid (Blackfish)	Long-finned pilot whale	7	Rifle 6.5x55	3			2
Delphinid (Blackfish)	Long-finned pilot whale	25	.30-06		23		
Delphinid (Blackfish)	Long-finned pilot whale	1	.303				
Delphinid (Blackfish)	Long-finned pilot whale	1	Shotgun	2			3
Delphinid (Blackfish)	Long-finned pilot whale	10	.30-06			10	
Delphinid (Blackfish)	Long-finned pilot whale	71	.303	3	70	1	
Delphinid (Blackfish)	Pilot whale (unknown sp.)	8	.308	3	5		3
Delphinid (Blackfish)	Pilot whale (unknown sp.)	30	.30-06	3	27		0.3
Delphinid (Blackfish)	Pilot whale (unknown sp.)	19	.303	1	19		
Delphinid (Blackfish)	Pygmy killer whale	7	.308	2		7	
Delphinid (Blackfish)	Pygmy killer whale	3	Unknown	3			
Delphinid (Blackfish)	Short-finned pilot whale	3	.30-06	1	3		
Kogiid	Pygmy sperm whale	1	.303	1			

Kogiid	Pygmy sperm whale	2	.30-06	3			1		
Kogiid	Pygmy sperm whale	1	.308	3			1		
Kogiid	Pygmy sperm whale	7	.308	3			7		
Kogiid	Pygmy sperm whale	1	.303	1	soft point	150 gm	1		
Kogiid	Pygmy sperm whale	2	.30-06	1	soft point	150 gm	2		
Kogiid	Pygmy sperm whale	6	.30-06		soft point	140 gm	6		
Kogiid	Pygmy sperm whale	1	.303		soft point	150 gm	1		
Kogiid	Pygmy sperm whale	3	.303				3		
Kogiid	Pygmy sperm whale	2	.308	1			2		
Kogiid	Pygmy sperm whale	1	.30-06				1		
Kogiid	Pygmy sperm whale	1	.308	1			1		
Kogiid	Pygmy sperm whale	2	.303	1			2		
Kogiid	Pygmy sperm whale	8	.30-06				8		
Kogiid	Pygmy sperm whale	2	.303				2		
Kogiid	Pygmy sperm whale	1	.270	2			1		
Kogiid	Pygmy sperm whale	1	.30-06				1		
Kogiid	Pygmy sperm whale	2	.303	1			2		
Kogiid	Pygmy sperm whale	4	.30-06	1			3		
Kogiid	Pygmy sperm whale	2	.303	1			2		
Kogiid	Pygmy sperm whale	2	.30-06				1	2	
Kogiid	Pygmy sperm whale	3	.303				2	1	
Kogiid	Pygmy sperm whale	1	.300	2				1	
Kogiid	Pygmy sperm whale	2	.303	1					
Mysticete	Bryde's whale	1	.270	1			1		
Mysticete	Dwarf minke whale	1	.30-06	3			1		
Mysticete	Humpback	1	.303						720
Mysticete	Humpback	1	.416	4					
Mysticete	Minke whale	1	.308	1			1		
Mysticete	Minke whale	1	.308	1			1		
Mysticete	Minke whale	1	.270	3					0.3

Mysticete	Minke whale	1	.30-06	3				1
Mysticete	Sei whale	1	.30-06	1			1	
Ziphiid	Arnoux's beaked whale	2	.303	1			2	
Ziphiid	Cuvier's beaked whale	1	Unknown					
Ziphiid	Cuvier's beaked whale	1	.30-06	1			1	
Ziphiid	Cuvier's beaked whale	1	.30-06	6				35
Ziphiid	Gray's beaked whale	1	.308	1			1	
Ziphiid	Gray's beaked whale	1	.44 magnum	1			1	
Ziphiid	Gray's beaked whale	1	.44 magnum	1			1	
Ziphiid	Gray's beaked whale	1	.303	2			1	
Ziphiid	Gray's beaked whale	1	.303	1			1	
Ziphiid	Strap-toothed whale	1	.308	2			1	
Ziphiid	Strap-toothed whale	1	.308	2	soft point	180 gm	1	

References

1. Arnould, J.P.Y.; Boyd, I.L.; Speakman, J.R. Measuring the Body Composition of Antarctic Fur Seals (Arctocephalus Gazella): Validation of Hydrogen Isotope Dilution. **1996**, *69*, 93–116, doi:10.1086/physzool.69.1.30164202.
2. Gosselin, J.; Boily, F. Unusual Southern Occurrence of a Juvenile Bearded Seal, Erignathus Barbatus, in the St Lawrence Estuary, Canada. *Marine Mammal Science* **1994**, *10*, 480–483.
3. Davis, D.A.; Mondo, K.; Stern, E.; Annor, A.K.; Murch, S.J.; Coyne, T.M.; Brand, L.E.; Niemeyer, M.E.; Sharp, S.; Bradley, W.G.; et al. Cyanobacterial Neurotoxin BMAA and Brain Pathology in Stranded Dolphins. *PLoS One* **2019**, *14*, e0213346–e0213346, doi:10.1371/journal.pone.0213346.
4. Dennison, S.; Gulland, F.; Haulena, M.; De Morais, H.; Colegrove, K. Urate Nephrolithiasis in a Northern Elephant Seal (Mirounga Angustirostris) and a California Sea Lion (Zalophus Californianus). *Journal of Zoo and Wildlife Medicine* **2007**, *38*, 114–20, doi:10.1638/05-121.1.
5. Mulsow, J.; Reichmuth, C. Electrophysiological Assessment of Temporal Resolution in Pinnipeds. *Aquatic Mammals* **2007**, *33*, 122–131, doi:10.1578/am.33.1.2007.122.
6. Dennison, S.; Haulena, M.; Williams, D.C.; Dawson, J.; Yandell, B.S.; Gulland, F.M.D. Determination of a Sedative Protocol for Use in California Sea Lions (Zalophus Californianus) with Neurologic Abnormalities Undergoing Electroencephalographic Examination. *Journal of Zoo and Wildlife Medicine* **2008**, *39*, 542–547, doi:10.1638/2007-0077.1.

7. Sarran, D.; Greig, D.J.; Rios, C.A.; Zabka, T.S.; Gulland, F.M.D. Evaluation of Aqueous Humor as a Surrogate for Serum Biochemistry in California Sea Lions (Zalophus Californianus). *Aquatic Mammals* **2008**, *34*, 157–165, doi:10.1578/am.34.2.2008.157.
8. Gulland, F.; Hall, A.; Greig, D.; Frame, E.; Colegrove, K.; Booth, R.; Wasser, S.; Scott-Moncrieff, J. Evaluation of Circulating Eosinophil Count and Adrenal Gland Function in California Sea Lions Naturally Exposed to Domoic Acid. *Journal of American Veterinary Medical Association* **2012**, *241*, 943–949.
9. Sawyer, E.K.; Turner, E.C.; Kaas, J.H. Somatosensory Brainstem, Thalamus, and Cortex of the California Sea Lion (Zalophus Californianus). *Journal of Comparative Neurology* **2016**, *524*, 1957–75, doi:10.1002/cne.23984.
10. Fauquier, D.A.; Gulland, F.M.D.; Trupkiewicz, J.G.; Spraker, T.R.; Lowenstine, L.J. Coccidioidomycosis in Free-Living California Sea Lions (Zalophus Californianus) in Central California. *Journal of Wildlife Diseases* **1996**, *32*, 707–710.
11. Fauquier, D.; Gulland, F.; Haulena, M.; Dailey, M.; Rietcheck, R.; Lipscomb, T. Meningoencephalitis in Two Stranded California Sea Lions (Zalophus Californianus) Caused by Aberrant Trematode Migration. *Journal of Wildlife Diseases* **2004**, *40*, 816–819.
12. Cameron, S.; Lopez, A.; Glabman, R.; Abrams, E.; Johnson, S.; Field, C.; Gulland, F.M.D.; Buckmaster, P.S. Proportional Loss of Parvalbumin-Immunoreactive Synaptic Boutons and Granule Cells from the Hippocampus of Sea Lions with Temporal Lobe Epilepsy. *Journal of Comparative Neurology* **2019**, *527*, 2341–2355, doi:10.1002/cne.24680.
13. De Graaf, A.; Shaughnessy, P.; McCully, R.; Verster, A. Occurrence of Taenia Solium in a Cape Fur Seal (Arctocephalus Pusillus). *Onderstepoort Journal of Veterinary Research* **1980**, *47*, 119–120.
14. Bernaldo de Quiros, Y.; Hartwick, M.; Rotstein, D.S.; Garner, M.M.; Bogomolni, A.; Greer, W.; Niemeyer, M.E.; Early, G.; Wenzel, F.; Moore, M. Discrimination between Bycatch and Other Causes of Cetacean and Pinniped Stranding. *Dis Aquat Organ* **2018**, *127*, 83–95, doi:10.3354/dao03189.
15. Glanville, E.; Bartlett, P.; Berrow, S. Common Dolphin Delphinus Delphis (L.). *The Irish Naturalists' Journal* **2003**, *27*, 241–242.
16. Kirkman, S.P.; Meÿer, M.A.; Thornton, M. False Killer Whale Pseudorca Crassidens Mass Stranding at Long Beach on South Africa ’s Cape Peninsula, 2009. *African Journal of Marine Science* **2010**, *32*, 167–170, doi:10.2989/1814232x.2010.481168.
17. Daoust, P.; Ortenburger, A. Successful Euthanasia of a Juvenile Fin Whale. *Canadian Veterinary Journal* **2001**, *42*, 127–129.
18. Dunn, J.L. Multiple-Agent Euthanasia of a Juvenile Fin Whale, Balanoptera Physalus. *Marine Mammal Science* **2006**, *22*, 1004–1007, doi:10.1111/j.1748-7692.2006.00073.x.
19. Dailey, M.; Gulland, F.; Lowenstine, L.J.; Silvagni, P.; Howard, D. Prey, Parasites and Pathology Associated with the Mortality of a Juvenile Gray Whale (Eschrichtius Robustus) Stranded along the Northern California Coast. *Diseases of Aquatic Organisms* **2000**, *42*, 111–117.
20. Gulland, F.; Pérez-Cortés, H.; Urban, J.; Rojas-Bracho, L.; Ylitalo, G.; Weir, J.; Norman, S.; M. Muto, M.; Rugh, D.; Johnson, C.; et al. *Eastern North Pacific Gray Whale (Eschrichtius Robustus) Unusual Mortality Event, 1999-2000*; US Dep. Commer. NOAA, 2005; p. 33;.
21. Frouin, H.; Ménard, L.; Measures, L.; Brousseau, P.; Fournier, M. T Lymphocyte-Proliferative Responses of a Grey Seal (Halichoerus Grypus) Exposed to Heavy Metals and PCBs in Vitro. *Aquatic Mammals* **2010**, *36*, 365–371, doi:10.1578/am.36.4.2010.365.
22. Sosa, M.; Gamble, K.C.; Delaski, K.; Righton, A. Clinical Challenge: Systemic Rhizopus Microsporus Infection with Renal Cavitation in a Grey Seal (Halichoerus Grypus). *Journal of Zoo and Wildlife Medicine* **2013**, *44*, 1134–8, doi:10.1638/2013-0089R.1.
23. Barnett, J.; Riley, P.; Cooper, T.; Linton, C.; Wessels, M. Mycotic Encephalitis in a Grey Seal (Halichoerus Grypus) Pup Associated with Rhizomucor Pusillus Infection. *Veterinary Record Case Reports* **2014**, *2*, doi:10.1136/vetreccr-2014-000115.
24. Silpa, M.A.C.; Thornton, S.M.; Cooper, T.; Hedley, J. Prevalence of Presenting Conditions in Grey Seal Pups (Halichoerus Grypus) Admitted for Rehabilitation. *Veterinary Science* **2015**, *2*, 1–11, doi:10.3390/vetsci2010001.
25. Stamper, M.; FMD, G.; Spraker, T. Leptospirosis in Rehabilitated Pacific Harbor Seals from California. *Journal of Wildlife Diseases* **1998**, *34*, 407–410.
26. Lapointe, J.; Gulland, F.; Haines, D.; Barr, B.; Duignan, P.J. Placentitis Due to Coxiella Burnetii in a Pacific Harbor Seal (Phoca Vitulina Richardsi). *Journal of Veterinary Diagnostic Investigation* **1999**, *11*, 541–543.

27. Dennison, S.E.; Forrest, L.J.; Fleetwood, M.L.; Gulland, F.M.D. Concurrent Occipital Bone Malformation and Atlantoaxial Subluxation in a Neonatal Harbor Seal (Phoca Vitulina). *Journal of Zoo and Wildlife Medicine* **2009**, *40*, 385–388.
28. Duignan, P.J.; Saliki, J.T.; Aubin, D.J.St.; Early, G.; Sadove, S.; House, J.A.; Kovacs, K.; Geraci, J.R. *EPIZOOTIOLOGY OF MORBILLIVIRUS INFECTION IN NORTH AMERICAN HARBOR SEALS (PHOCA VITULINA) AND GRAY SEALS (HALICHOERUS GRYPUS)*; BIOONE, 1995; Vol. 31;.
29. Bodewes, R.; Rubio García, A.; Wiersma, L.C.M.; Getu, S.; Beukers, M.; Schapendonk, C.M.E.; van Run, P.R.W.A.; van de Bildt, M.W.G.; Poen, M.J.; Osinga, N.; et al. Novel B19-Like Parvovirus in the Brain of a Harbor Seal. *PLOS ONE* **2013**, *8*, e79259, doi:10.1371/journal.pone.0079259.
30. Flower, J.E.; Gamble, K.C.; Stone, M.; Lyons, J.A.; Maganti, R.J.; Tuomi, P.A.; Olds, J.E.; Sims, M.A.; Gauger, P.; Tuttle, A.D. Esophageal Squamous Cell Carcinoma in Six Harbor Seals (Phoca Vitulina Spp.). *Journal of Zoo and Wildlife Medicine* **2014**, *45*, 620–31, doi:10.1638/2012-0218R1.1.
31. Bodewes, R.; Hapsari, R.; Rubio Garcia, A.; Sanchez Contreras, G.J.; van de Bildt, M.W.; de Graaf, M.; Kuiken, T.; Osterhaus, A.D. Molecular Epidemiology of Seal Parvovirus, 1988-2014. *PLoS One* **2014**, *9*, e112129, doi:10.1371/journal.pone.0112129.
32. Daoust, P.; Haines, D.; Thorsen, J.; Duignan, P.J.; Geraci, JR. Phocine Distemper in a Harp Seal (Phoca Groenlandica) from the Gulf of St. Lawrence, Canada. *Journal of Wildlife Diseases* **1993**, *29*, 114–117.
33. Desforges, J.P.; Levin, M.; Jasperse, L.; De Guise, S.; Eulaers, I.; Letcher, R.J.; Acquarone, M.; Nordoy, E.; Folkow, L.P.; Hammer Jensen, T.; et al. Effects of Polar Bear and Killer Whale Derived Contaminant Cocktails on Marine Mammal Immunity. *Environmental Science Technology* **2017**, *51*, 11431–11439, doi:10.1021/acs.est.7b03532.
34. Sonne, C.; Andersen-Ranberg, E.; Rajala, E.L.; Agerholm, J.S.; Bonefeld-Jorgensen, E.; Desforges, J.P.; Eulaers, I.; Jenssen, B.M.; Koch, A.; Rosing-Asvid, A.; et al. Seroprevalence for Brucella Spp. in Baltic Ringed Seals (Phoca Hispida) and East Greenland Harp (Pagophilus Groenlandicus) and Hooded (Cystophora Cristata) Seals. *Veterinary Immunology and Immunopathology* **2018**, *198*, 14–18, doi:10.1016/j.vetimm.2018.02.005.
35. Larsen, A.K.; Nymo, I.H.; Boysen, P.; Tryland, M.; Godfroid, J. Entry and Elimination of Marine Mammal Brucella Spp. by Hooded Seal (Cystophora Cristata) Alveolar Macrophages in Vitro. *PLoS One* **2013**, *8*, e70186, doi:10.1371/journal.pone.0070186.
36. Bischoff, K.; Jaeger, R.; Ebel, J.G. An Unusual Case of Relay Pentobarbital Toxicosis in a Dog. *J Med Toxicol* **2011**, *7*, 236–239, doi:10.1007/s13181-011-0160-8.
37. Harms, C.A.; McLellan, W.A.; Moore, M.J.; Barco, S.G.; Clarke, E.O., 3rd; Thayer, V.G.; Rowles, T.K. Low-Residue Euthanasia of Stranded Mysticetes. *Journal of Wildlife Diseases* **2014**, *50*, 63–73, doi:10.7589/2013-03-074.
38. Glezer, I.; Morgane, P. Ultrastructure of Synapses and Golgi Analysis of Neurons in Neocortex of the Lateral Gyrus (Visual Cortex) of the Dolphin and Pilot Whale. *Brain Research Bulletin* **1990**, *24*, 401–427.
39. White, R.; Shea, D.; Schlezinger, J.; Hahn, M.; Stegeman, J. In Vitro Metabolism of Polychlorinated Biphenyl Congeners by Beluga Whale (Delphinapterus Leucas) and Pilot Whale (Globicephala Melas) and Relationship to Cytochrome P450 Expression. *Comparative Biochemistry and Physiology* **2000**, *126*, 267–284.
40. Brownlow, A.; Baily, J.; Dagleish, M.; Deaville, R.; Foster, G.; Jensen, S.; Krupp, E.; Law, R.; Penrose, R.; Perkins, M.; et al. *Investigation into the Long-Finned Pilot Whale Mass Stranding Event, Kyle of Durness, 22nd July 2011*; Department for Environment Food & Rural Affairs (DEFRA) Scottish Marine Animal Stranding Scheme; Scotland, 2015;
41. Phillips, A.C.N.; Suepaul, R. Nasitrema Species: A Frequent Culprit in Melon-Headed Whale (Peponocephala Electra) Strandings in Trinidad. *Aquatic Mammals* **2017**, *43*, 547–557, doi:10.1578/am.43.5.2017.547.
42. Tarpley, R.; Marwitz, S. Plastic Debris Ingestion by Cetaceans along the Texas Coast Two Case Reports. *Aquatic Mammals* **1993**, *19.2*, 93–98.
43. Hohn, A.A.; Rotstein, D.S.; Harms, C.A.; Southall, B.L. *Multispecies Mass Stranding of Pilot Whales (Globicephala Macrorhynchus), Minke Whale (Balaenoptera Acutorostrata), and Dwarf Sperm Whales (Kogia Sima) in North Carolina on 15-16 January 2005*; Report on Marine Mammal Unusual Mortality Event UMESE0501Sp; NOAA, 2006;
44. Harms, C.A.; Lovewell, G.N.; Rotstein, D.S. Presumed Hyperglycemic Cataracts in a Live-Stranded Minke Whale (Balaenoptera Acutorostrata) Calf. *Marine Mammal Science* **2008**, *24*, 388–397, doi:10.1111/j.1748-7692.2007.00182.x.

45. Donahoe, S.L.; Rose, K.; Slapeta, J. Multisystemic Toxoplasmosis Associated with a Type II-like Toxoplasma Gondii Strain in a New Zealand Fur Seal (Arctocephalus Forsteri) from New South Wales, Australia. *Veterinary Parasitology* **2014**, *205*, 347–53, doi:10.1016/j.vetpar.2014.07.022.
46. Gulland, F.M.D.; Werner, L.; O'Neill, S.; Lowenstine, L.J.; Trupkiewitz, J.; Smith, D.; Royal, B.; Strubel, I. Baseline Coagulation Assay Values for Northern Elephant Seals (Mirounga Angustirostris), and Disseminated Intravascular Coagulation in This Species. *Journal of Wildlife Diseases* **1996**, *32*, 536–540.
47. Trupkiewicz, J.G.; Gulland, F.M.D.; Lowenstine, L.J. Congenital Defects in Northern Elephant Seals Stranded along the Central California Coast. *Journal of Wildlife Diseases* **1997**, *33*, 220–225.
48. Haulena, M.; Buckles, E.; Gulland, F.; Lawrence, J.; Wong, A.; Jang, S.; Christopher, M.; Lowenstine, L. Systemic Mycosis Caused by Scedosporium Apiospermum in a Stranded Northern Elephant Seal (Mirounga Angustirostris) Undergoing Rehabilitation. *Journal of Zoo and Wildlife Medicine* **2002**, *33*, 166–171, doi:10.1638/1042-7260(2002)033[0166:Smcbsa]2.0.Co;2.
49. Fauquier, D.; Gulland, F.; Haulena, M.; Spraker, T. Biliary Adenocarcinoma in a Stranded Northern Elephant Seal (Mirounga Angustirostris). *Journal of Wildlife Diseases* **2003**, *39*, 723–726.
50. Turner, E.C.; Sawyer, E.K.; Kaas, J.H. Optic Nerve, Superior Colliculus, Visual Thalamus, and Primary Visual Cortex of the Northern Elephant Seal (Mirounga Angustirostris) and California Sea Lion (Zalophus Californianus). *J Comp Neurol* **2017**, *525*, 2109–2132, doi:10.1002/cne.24188.
51. Elson-Riggins, J.G.; Al-Banna, L.; Platzer, E.G.; Kaloshian, I. Characterization of Otostrongylus Circumlitus from Pacific Harbor and Northern Elephant Seals. *Journal of Parasitology* **2001**, *87*, 73–78, doi:10.1645/0022-3395(2001)087[0073:Coocfp]2.0.Co;2.
52. Farley, S.D.; Robbins, C.T. Development of Two Methods to Estimate Body Composition of Bears. *Canadian Journal of Zoology* **1994**, *72*, 220–226, doi:10.1139/z94-029.
53. Clua, E.; Manire, C.; Garrigue, C. Biological Data of Pygmy Killer Whale (Feresa Attenuata) from a Mass Stranding in New Caledonia (South Pacific) Associated with Hurricane Jim in 2006. *Aquatic Mammals* **2014**, *40*, 162–172, doi:10.1578/AM.40.2.2014.162.
54. Rousselet, E.; Stolen, M.; Durden, W.N.; Jablonski, T.; Stacy, N.I.; Rotstein, D.S. Bilateral Polycystic Kidneys and Focal Renal Cystadenoma in a Pygmy Sperm Whale (Kogia Breviceps). *Journal of Wildlife Diseases* **2019**, *55*, 258–261, doi:10.7589/2018-01-019.
55. Kolesnikovas, C.; Groch, K.; Groch, K.; Moraes, A.; Flores, P.; Pretto, D.; Freitas, R.; Gaidzinski, M.; Moreira, L. Euthanasia of an Adult Southern Right Whale (Eubalaena Australis) in Brazil. *Aquatic Mammals* **2012**, *38*, 317–321, doi:10.1578/am.38.3.2012.317.
56. Miller, M.; Conrad, P.; James, E.R.; Packham, A.; Toy-Choutka, S.; Murray, M.J.; Jessup, D.; Grigg, M. Transplacental Toxoplasmosis in a Wild Southern Sea Otter (Enhydra Lutris Nereis). *Veterinary Parasitology* **2008**, *153*, 12–8, doi:10.1016/j.vetpar.2008.01.015.
57. St Leger, J.A.; Righton, A.L.; Nilson, E.M.; Fascetti, A.J.; Miller, M.A.; Tuomi, P.A.; Goertz, C.E.; Puschner, B. Vitamin A Deficiency and Hepatic Retinol Levels in Sea Otters, Enhydra Lutris. *Journal of Zoo and Wildlife Medicine* **2011**, *42*, 98–104, doi:10.1638/2010-0010.1.
58. Peterson, J.C.; Hoggard, W. First Sperm Whale (Physeter Macrocephalus) Record in Mississippi. *Gulf Research Reports* **1996**, *9*, doi:10.18785/grr.0903.10.
59. Thayer, V.G.; Harms, C.A.; Rittmaster, K.A.; Rotstein, D.S.; Hairr, J.E. A North Carolina Stranding of a White-Beaked Dolphin (Lagenorhynchus Albirostris), Family Delphinidae: A New Southerly Record. *Aquatic Mammals* **2018**, *44*, 32–38, doi:10.1578/am.44.1.2018.32.
60. Reichmuth, C.; Mulsow, J.; Finneran, J.J.; Houser, D.S.; Supin, A.Y. Measurement and Response Characteristics of Auditory Brainstem Responses in Pinnipeds. *Aquatic Mammals* **2007**, *33*, 132–150, doi:10.1578/am.33.1.2007.132.
61. Blackmore, D.K.; Madie, P.; Bowling, M.C.; Nutman, A.; Davies, A.S.; McLeod, W.R.; Taylor, J.; Degen, M. The Use of a Shotgun for Euthanasia of Stranded Cetaceans. *New Zealand Veterinary Journal* **1995**, *43*, 158–9, doi:10.1080/00480169.1995.35878.
62. Stockin, K.A.; Duignan, P.J.; Roe, W.D.; Meynier, L.; Alley, M.; Fettermann, T. Causes of Mortality in Stranded Common Dolphin (Delphinus Sp.) from New Zealand Waters between 1998 and 2008. *Pacific Conservation Biology* **2009**, *15*, 217–227.
63. Coughran, D.K.; Stiiles, I.; Mawson, P. Euthanasia of Beached Humpback Whales Using Explosives. *Journal of Cetacean Research and Management* **2012**, *12*, 137–144.

64. Ogle, M. Managing the Welfare of Marine Mammals at Mass Strandings in Golden Bay, New Zealand. In *Marine Mammal Welfare*; Animal Welfare: ; Springer International Publishing: New York, USA; 2017; pp. 137–146 ISBN 978-3-319-46993-5.
65. Hunter, S.; Ogle, R.; Kirk, E. The Mass Stranding Event of Long-Finned Pilot Whales, Globocephalus Melas, at Golden Bay in February 2017. *Kokako* **2017**, *24*, 27–36.
66. Hampton, J.; Mawson, P.; Coughran, D.; Vitali, S. Validation of the Use of Firearms for Euthanising Stranded Cetaceans. *Journal of Cetacean Research Management* **2014**, *14*, 117–123.
